# Benefits of patient risk stratification and targeted interventions on multidrug resistant pathogens prevention and control

**DOI:** 10.1007/s44250-022-00006-6

**Published:** 2022-11-14

**Authors:** Emanuele Russo, Silvia Di Bari, Vanni Agnoletti, The local ICU Infection Control Team, Marinella Bagni, Barbara Bertaccini, Deborah Campagna, Arianna Giacomini, Elisa Magalotti, Federica Marson, Consuelo Morena, Valentina Muccioli, Giulia Paci, Maria Andrea Palazzo, Erika Pirini, Alice Rasi, Maurizio Ravaldini, Giulia Sauchelli, Martina Spiga, Claudia Turrini

**Affiliations:** 1 Anesthesia and Intensive Care Unit, Bufalini Hospital, AUSL della Romagna, Cesena, Italy; 2 Anesthesia and Intensive Care Department, Alma Mater Studiorum - Università di Bologna, Azienda Ospedaliero-Universitaria di Bologna IRCCS, via Giuseppe Massarenti 9, 40138 Bologna, Italy

**Keywords:** Infection control, Antimicrobial Stewardship, Multidrug resistance, Contact precautions, Intensive Care Unit, Infection prevention, Hospital infection control, Cross infection prevention

## Introduction

Antimicrobial resistance (AMR) is considered a major global health problem and location-specific policy are warranted with urgent priority. A recent systematic analysis has estimated 4.95 million deaths worldwide associated with bacterial AMR in 2019 [1].

AMR has especially highlighted important infection control implications in the Intensive Care Unit (ICU) settings where multidrug resistant (MDR) organisms are being isolated with growing frequency [2].

The most threatening MDR bacteria are methicillin-resistant *Staphylococcus* *aureus* (MRSA), vancomycin-resistant enterococci (VRE), *Acinetobacter* *baumannii*, Enterobacteriaceae that produce extended-spectrum beta-lactamases and/or carbapenemases (eg, ESBLs and CREs, respectively), and carbapenem-resistant *Pseudomonas* *aeruginosa*, along with emerging MDR fungal pathogens, with *Candida* *auris* currently menacing ICUs worldwide [3].

European Antimicrobial Resistance Surveillance Network (EARS-Net), coordinated by European Centre for Disease Prevention and Control, found that the overall number of reported isolates at EU/EEA level increased in 2020 compared to 2019 for almost all bacterial species, with Italy having worrisome and statistically significant increasing trends in Acinetobacter spp., VRE and CRE isolation [4].

The National Healthcare Safety Network (NHSN), United States’ largest electronic surveillance system for tracking healthcare-associated infections managed by the Centers for Disease Control and Prevention (CDC), reported in their latest summary statistically significant differences in the percentages of pathogens with nonsusceptibility to selected antimicrobials between hospital wards and ICUs, with ICUs tending to have a higher resistance rate for bloodstream infections [5].

Prevention and control of MDR organisms require an extensive financial and human resource commitment, along with specific scientific knowledge and administrative leadership, in order to be successful.

Although some encouraging new antimicrobials have been approved, the number of novel compounds with a new mechanism of action remains small [6]. While new interventions are being developed, such as immunotherapy, prevention through infection control practices needs to be maximized [7].

Improvement in prevention strategies might especially prove helpful in low-resource health systems, where access to antibiotics is limited and nonetheless the highest burden due to AMR was found [1].

## Infection control strategies

Strategies to prevent the emergence and spread of MDR pathogens in ICUs can be divided into two major categories: antibiotic stewardship (AS) (to reduce selective pressure) [8] and infection prevention and control measures (IPC) (to reduce colonization pressure) [9, 10].

IPC was defined by the World Health Organization as an evidence-based approach which attempts to prevent transmission of avoidable infections, including those caused by MDR, acquired during the provision of health care services [11]. Such interventions can lower the risk of spread by up to 70% when effectively applied [12]. Standard measures in the ICUs consist of hand hygiene, contact precautions (CPs), cohorting and dedicated staff, decolonization and daily bathing with chlorhexidine, digestive decontamination, environmental cleaning, surveillance and device-specific strategies [13]. Multimodal programs were found to be more effective [14].

## Risk factors, risk stratification

Although appropriate antibiotic therapy administration has contributed to reduce selective pressure [15], by limiting the emergence of MDR strains, colonization pressure still plays a major role in ICUs due to cross-transmission from colonized patient or environmental reservoirs. Colonization pressure, defined as the ratio of patients colonized with MDR relative to all patients, has been reported to be independently associated with ICU-acquired MDR related infections [16]. Such finding has the clinical implication of stressing the importance of screening patients at admission time and cohorting MDR carriers.

Many risk factors have been linked to the likelihood of MDR carriage and/or infection, the common ones being prior exposure to antibiotics, prolonged hospital and ICU length of stay, indwelling devices, comorbidities and local epidemiology [3, 17–19]. However, most authors do not differentiate between risk factors for prior MDR colonization and risk factors for new acquisition. Burillo et al. in their review clearly made the distinction and searched the evidence for predictive scores of MDR Gram-negative carriers [18]. They found that only one model to detect colonization at hospital admission was developed by Tseng et al. [20]. The complex links and interactions between environment, microbial behaviour and patients’ characteristics, however, explain why no unified risk stratification of MDR colonization on admission in either hospitals or ICUs has ever been designed [18, 21].

## Pre-emptive isolation and our approach

Empiric isolation is meant for symptomatic cases in which a transmissible infectious disease is suspected but tests have not yet confirmed it. Such strategy is largely adopted in common infectious diseases where symptoms clearly ring the bell of possible diffusion, like contact precautions in diarrhoea or droplet isolation in influenza [13]. By contrast, to our knowledge neither common practice nor clear recommendation about preventive contact isolation in “silent” colonization seem to exist, which affects especially MDR management rather than common pathogens. In 2006, however, the CDC’s Guideline for Isolation Precautions had to be integrated with a specific document on MDR prevention as new concerns about increasing incidence were raised [22]. In fact, they recommend a set of intensified MDR prevention interventions to be applied when standard measures are not sufficient, with the important adjunctive of individualizing practice according to local considerations [2]. Preventive contact isolation of new admissions pending results of screening cultures are reported in their review, but no formal recommendation has been formulated. Nevertheless, we believe that such finding is representative of widespread concerns about subtle MDR colonization and the need for pre-emptive strategies.

In the present paper, we describe a single-centre strategy with the aim of raising some interesting cause for reflection on ICU patient management as far as AMR is concerned. The following strategy refers to a polyvalent Bufalini Hospital ICU (Cesena, Italy), consisting of 23 beds organized into three multibed rooms, one double-bed room and three single-bed rooms. The ICU admits an average of 1,000 patients per year, 280 of which are trauma patients and about 500 are surgical (both emergency and elective). Besides accurate antimicrobial stewardship program [8] and common infection control interventions, a precautionary policy was established as an adjunctive standard measure. According to it, the probability of a patient being colonized/infected by MDR organisms is assessed as soon as we receive a transfer notification, by collecting anamnestic and previous laboratory data. When this is not feasible, assessment occurs on admission. Risk factors taken into account on ICU admission are: MDR patient colonization either known on admission time or in the previous 6 months; transfer from a ward/ICU with a high MDR rate; hospitalization for more than 7 days; transfer from a rehabilitation or long-term facility; broad spectrum antibiotic therapy for more than seven days during the previous thirty days. As ICU stay continues, the patient’s risk factors undergo changes and are therefore daily re-assessed. Daily reassessment of MDR colonization/infection risk is performed at the time of the daily multidisciplinary briefing attended by intensivists, nurses, case managers, infectivologists, physical therapists, physiatrists, perfusionists and psychologists; MDR risk assessment is deemed to be as relevant as the other clinical and laboratory data. All risk factors are assessed for each patient. The most important ICU-related risk factors we evaluate are broad spectrum antimicrobial therapy for more than seven days; ICU stay for more than 15 days; newly acquired MDR colonization or MDR selection in an infected patient. According to risk stratification, additional CPs are introduced including CPs, optimizing nurse-patient ratio of 1:1 and patient isolation in individual room. Along with additional CPs, we regularly apply standard measures with particular regard to pondering the need for invasive devices and antibiotic therapy on a daily basis. Whenever possible, we choose to avoid empiric therapy and wait for cultures to start a targeted treatment. The need for empiric antibiotic therapy is established by clinical judgment based on the severity and site of infection, the patient's actual condition and comorbidities. In case an empiric antibiotic therapy is needed, the molecule choice is based on a complex and thorough evaluation encompassing: infection site and its typical pathogenic germs, odds of those germs being resistant to common empiric therapy, previous cultural tests, medical history, clinical conditions and previous antibiotic therapy. The prescription of antimicrobial therapy also follows a logic of clinical and MDR risk stratification. Every effort is made so that MDR selective pressure is minimized [23, 24].

Although such a precautionary policy is well known through hospitals, in literature only a few authors described in detail their protocols. Among these, Abella Álvarez et al. [25] found that the criteria used to detect MDR carriers led to an incidence of 69% false positive. Djibré et al. [21] compared targeted to universal additional CPs and found the former one to be noninferior. However, the risk factors for MDR carriers they tested did not prove a high positive predictive value. Interestingly, both works used more liberal criteria to determine MDR carriage, while in our protocol narrower and more selective risk factors are applied. Similar methods are regularly applied in a Spanish polyvalent ICU [26]. Like all the above authors, we think too that implementation of IPC programs is needed while knowledge and common practice have to be shared as much as possible in order to reach full understanding of the issue.

## Discussion

In this paper we describe our current strategy of placing patients under pre-emptive contact precautions whenever specific risk factors identifies possible MDR colonization, followed by constant monitoring for events that might change the assessment.

Since universal isolation seems unrealistic, ineffective and in certain cases detrimental, scrupulous patient selection is warranted [21, 27]. The same logic stands behind surveillance and screening, as thorough sampling is reckoned inappropriate and not feasible. As universal MDR screening in unjustified because of costs and individual burdens, specific screening on targeted population has been advocated [28]. We hold this to be true also for preventive measures. At the same time, the hazards of cross-transmission need to be minimized pending screening results. By applying a systematic and evidence-based risk stratification, patients with a high chance of MDR carriage can promptly be identified and targeted strategies adopted. In this regard, we believe that differentiating between the risk factors specific for prior colonization and those for new acquisition of MDR organisms in ICUs might help outline predictors-based containment/prevention programs.

On the other hand, patients not needing unnecessary isolation and contact precautions are spared, thus facilitating nurses’ assistance and medical care. For instance, during SARS-CoV-19 outbreak, the augmented use of personal protective equipment was supposed to cause higher levels of blood culture contaminations, which in turn spoils correct diagnosis and treatment [29]. Other reported adverse effects regard costs, reduced interaction between the healthcare professionals and patients, anxiety and depression [25]. Rubin et al. [30], however, argued that the highest quality evidence counter with a consistent worsening of human aspects. Rather, enhancement of IPC programs with institution of risk stratification protocols would have the effect of increasing health care workers awareness and knowledge about cross-transmission, which in turn leads to conscious participation, better compliance and lower infection rates [31, 32].

We regard monitoring as another key point in IPC. Prevention strategies must be followed by adequate and constant monitoring in order to critically judge their benefits and their disadvantages. Clinic, radiology, procalcitonin trend, surveillance cultures and periodic score calculations are ready-to-use tools but have some predictive limitations. Monitoring local epidemiology through periodic surveillance and systematic screening of new admissions, as well as monitoring afferent wards’ epidemiology, has the double advantage of guiding antimicrobial stewardship and of proving IPC programs’ effectiveness. Mathematical models like transmission coefficient (i.e. rate at which a pathogen moves from infected individuals to susceptible individuals) were designed for the purpose [33, 34]. Authors warn, however, that they do not perfectly suit stochastic behaviour of certain diseases and therefore require large numbers to be estimated [35]. Predicting and verifying transmission rates still remains paramount to assess the force of infection and the response to the applied IPC interventions. Again, multimodality seems the best way to reach a thorough point of view.

Despite lack of evidence, we also advocate that IPC interventions should be customized in respect of certain pathogens once they are confirmed. It is well known, in fact, that some MDR organisms such as Candida spp. and Acinetobacter have the ability to adhere to surfaces by means of biofilm formations [36, 37]. Not only this implies augmented AMR issues, but it also raises awareness on the need for specific devices and environmental strategies. For instance, *Pseudomonas* *aeruginosa* biofilm was found to be significantly less susceptible to disinfection by two oxidative biocides [38]. Yu et al. suggested the importance of disinfecting external ventilator circuits in order to prevent ventilator associated pneumonia and subsequent cross-transmission of MDR [39]. We could mention other examples, but the purpose of this paper is not to provide a complete description of the specific features of each pathogen. Research is actively making efforts to develop novel devices and materials [40], but until then IPC programs cannot ignore pathogens’ specificities. In our opinion, from pre-emptive and empiric to directed treatments, every aspect of IPC should undergo a process of “pathogen-tailoring”.

Testing the effectiveness of different cross-colonization control strategies requires studies with large population size, rigorous screeening measures implementation and a large amount of data on individual patient characteristics and risk factors. This paper is intended to be a call for research on the topic, starting of course with the results of our center.

## Conclusions

The purpose of this brief report is to illustrate the logic behind a pre-emptive and risk factors-based approach to potential MDR carriers. Aside from sharing knowledge and practices, we also intend to underline how these strategies need to be individualized according to local epidemiology, resources and patient’s characteristics. The importance of developing risk stratification tools has the purpose of keeping CPs as proportionate as possible, in opposition to a blind policy of massive and non-selective screening and isolation. Advantages of a reasoned and customized attitude include costs containment, decreased unnecessary stress to patients and reduced workload for personnel. The final effect would be a beneficial virtuous cycle where effective contact isolation leads to less need for isolation by means of keeping MDR rates low, while healthcare workers’ satisfaction and understanding would lead to even more efficacy by encouraging them to accurately observe protocols.

## Data Availability

Data sharing not applicable to this article as no datasets were generated or analysed during the current study.

## References

[1] Antimicrobial Resistance Collaborators. Global burden of bacterial antimicrobial resistance in 2019: a systematic analysis. Lancet. 2022;399(10325):629–55. 35065702 10.1016/S0140-6736(21)02724-0 PMC8841637

[2] Siegel JD, Rhinehart E, Jackson M, Chiarello L, Committee the HICPA. Management of multidrug-resistant organisms in healthcare settings, 2006. Cent Dis Control Prev. 2018;61(1):26–35. 10.1016/j.ajic.2007.10.006 18068814

[3] Lone SA, Ahmad A. *Candida**auris*—the growing menace to global health. Mycoses. 2019;62(8):620–37. 30773703 10.1111/myc.12904

[4] European Centre for Disease Prevention and Control. Antimicrobial resistance in the EU/EEA (EARS-Net) - Annual Epidemiological Report 2020. Stockholm: ECDC. 2022. https://www.ecdc.europa.eu/sites/default/files/media/en/aboutus/governance/competent-bodies/Documents/coordinating. Accessed 30 Jul 2022.

[5] Weiner-Lastinger LM, Abner S, Edwards JR, Kallen AJ, Karlsson M, Magill SS, et al. Antimicrobial-resistant pathogens associated with adult healthcare-associated infections: Summary of data reported to the National Healthcare Safety Network, 2015–2017. Infect Control Hosp Epidemiol. 2020;41(1):1–18. 31767041 10.1017/ice.2019.296 PMC8276252

[6] Boucher HW, Talbot GH, Bradley JS, Edwards JE, Gilbert D, Rice LB, et al. Bad bugs, no drugs: No ESKAPE! An update from the Infectious Diseases Society of America. Clin Infect Dis. 2009;48(1):1–12. 19035777 10.1086/595011

[7] Bassetti M, Poulakou G, Ruppe E, Bouza E, Van Hal SJ, Brink A. Antimicrobial resistance in the next 30 years, humankind, bugs and drugs: a visionary approach. Intensive Care Med. 2017;43(10):1464–75. 28733718 10.1007/s00134-017-4878-x

[8] Mandelli G, Dore F, Langer M, Garbero E, Alagna L, Bianchin A, et al. Effectiveness of a Multifaced Antibiotic Stewardship Program: A Pre-Post Study in Seven Italian ICUs. J Clin Med. 2022;11(15):4409. 35956026 10.3390/jcm11154409 PMC9369193

[9] Bonten MJM, Slaughter S, Ambergen AW, Hayden MK, Van Voorhis J, Nathan C, et al. The role of “colonization pressure” in the spread of vancomycin-resistant enterococci: an important infection control variable. Arch Intern Med. 1998;158(10):1127–32. 9605785 10.1001/archinte.158.10.1127

[10] Merrer J, Santoli F, De VCA, Tran B, De JB, Outin H. Colonization pressure and risk of acquisition of Methicillin-resistant *Staphylococcus**aureus*. Soc Healthc Epidemol Am. 2014;21(11):1–7. 10.1086/501721 11089656

[11] Core Competencies for Infection Prevention and Control. Geneva: World Health Organization; 2020. https://www.who.int/publications/i/item/9789240011656. Accessed 23 Aug 2022.

[12] Shankar Balakrishnan V. Newsdesk WHO’s first global infection prevention and control report. 2022. www.thelancet.com/infection. Accessed 5 Aug 2022. 10.1016/S1473-3099(22)00459-5 PMC9299724 35870460

[13] Strich JR, Palmore TN. Preventing transmission of multidrug-resistant pathogens in the intensive care unit. Infect Dis Clin North Am. 2017;31(3):535. 28687211 10.1016/j.idc.2017.05.010 PMC5584576

[14] Teerawattanapong N, Kengkla K, Dilokthornsakul P, Saokaew S, Apisarnthanarak A, Chaiyakunapruk N. Prevention and control of multidrug-resistant Gram-Negative bacteria in adult intensive care units: a systematic review and network meta-analysis. Clin Infect Dis. 2017;64(suppl_2):S51-60. 28475791 10.1093/cid/cix112

[15] Rice LB. Antimicrobial stewardship and antimicrobial resistance. Med Clin North Am. 2018;102(5):805–18. 30126572 10.1016/j.mcna.2018.04.004

[16] Masse J, Elkalioubie A, Blazejewski C, Ledoux G, Wallet F, Poissy J, et al. Colonization pressure as a risk factor of ICU-acquired multidrug resistant bacteria: a prospective observational study. Eur J Clin Microbiol Infect Dis. 2017;36(5):797–805. 28000030 10.1007/s10096-016-2863-x

[17] Safdar N, Maki DG. The commonality of risk factors for nosocomial colonization and infection with antimicrobial-resistant *Staphylococcus**aureus*, Enterococcus, Gram-Negative Bacilli, *Clostridium**difficile*, and Candida. Ann Intern Med. 2002;136:834–44. 12044132 10.7326/0003-4819-136-11-200206040-00013

[18] Burillo A, Muñoz P, Bouza E. Risk stratification for multidrug-resistant Gram-negative infections in ICU patients. Curr Opin Infect Dis. 2019;32(6):626–37. 31567570 10.1097/QCO.0000000000000599

[19] Lakhundi S, Zhang K. Methicillin-resistant *Staphylococcus**aureus*: molecular characterization, evolution, and epidemiology. Clin Microbiol Rev. 2018. 10.1128/CMR.00020-18. 30209034 10.1128/CMR.00020-18 PMC6148192

[20] Tseng WP, Chen YC, Yang BJ, Chen SY, Lin JJ, Huang YH, et al. Predicting multidrug-resistant Gram-Negative bacterial colonization and associated infection on hospital admission. Infect Control Hosp Epidemiol. 2017;38(10):1216–25. 28870265 10.1017/ice.2017.178

[21] Djibré M, Fedun S, Le Guen P, Vimont S, Hafiani M, Fulgencio JP, et al. Universal versus targeted additional contact precautions for multidrug-resistant organism carriage for patients admitted to an intensive care unit. Am J Infect Control. 2017;45(7):728–34. 10.1016/j.ajic.2017.02.001. 28285725 10.1016/j.ajic.2017.02.001

[22] Siegel JD, Rhinehart E, Jackson M, Chiarello L, the Healthcare Infection Control Practices Advisory Committee. 2007 Guideline for isolation precautions: preventing transmission of infectious agents in healthcare settings. https://www.cdc.gov/infectioncontrol/guidelines/isolation/index.html. Accessed 30 July 2022. 10.1016/j.ajic.2007.10.007 PMC7119119 18068815

[23] Hranjec T, Rosenberger LH, Swenson B, Metzger R, Flohr TR, Politano AD, et al. Aggressive versus conservative initiation of antimicrobial treatment in critically ill surgical patients with suspected intensive-care-unit-acquired infection: a quasi-experimental, before and after observational cohort study. Lancet Infect Dis. 2012;12(10):774–80. 22951600 10.1016/S1473-3099(12)70151-2 PMC3462590

[24] Hranjec T, Sawyer RG. Conservative initiation of antimicrobial treatment in ICU patients with suspected ICU-acquired infection: more haste less speed. Curr Opin Crit Care. 2013;19(5):461–4. 23995121 10.1097/MCC.0b013e328364d525 PMC3888949

[25] Abella Álvarez A, Janeiro Lumbreras D, Lobo Valbuena B, Naharro Abellán A, Torrejón Pérez I, Enciso Calderón V, et al. Analysis of the predictive value of preventive isolation criteria in the intensive care unit. Med Intensiva (English Ed). 2021;45(4):205–10. 10.1016/j.medin.2019.09.022 31780256

[26] Álvarez Lerma F, Granado Solano J, García Sanz A, López Martínez C, Herrera Sebastián R, Salvat Cobeta C, et al. Optimization of pre-emptive isolations in a polyvalent ICU through implementation of an intervention strategy. Med Intensiva (English Ed). 2015;39(9):543–51. 10.1016/j.medin.2014.11.010 25798954

[27] Furuya EY, Cohen B, Jia H, Larson EL. Long-term impact of universal contact precautions on rates of multidrug-resistant organisms in ICUs: a comparative effectiveness study. Infect Control Hosp Epidemiol. 2018;72(23):2964–79. 10.1017/ice.2018.35 PMC5935260 29562944

[28] Nijsingh N, Munthe C, Lindblom A, Åhrén C. Screening for multi-drug-resistant Gram-negative bacteria: what is effective and justifiable? Monash Bioeth Rev. 2020;38(1):72–90. 10.1007/s40592-020-00113-1. 32356217 10.1007/s40592-020-00113-1 PMC7749868

[29] Russo E, Bolondi G, Gamberini E, Santonastaso DP, Circelli A, Spiga M, et al. Increased blood culture contamination rate during COVID-19 outbreak in intensive care unit: A brief report from a single-centre. J Intensive Care Soc. 2021;23(4):17511437211012152. 10.1177/17511437211012152 PMC9679905 36751343

[30] Rubin MA, Samore MH, Harris AD. The importance of contact precautions for endemic methicillin-resistant *Staphylococcus**aureus* and vancomycin-resistant enterococci. J Am Med Assoc. 2018;319(9):863–4. 10.1001/jama.2017.21122 29435582

[31] Kasatpibal N, Chittawatanarat K, Nunngam N, Kampeerapanya D, Duangsoy N, Rachakom C, et al. Impact of multimodal strategies to reduce multidrug-resistant organisms in surgical intensive care units: Knowledge, practices and transmission: a quasi-experimental study. Nurs Open. 2021;8(4):1937. 33760380 10.1002/nop2.864 PMC8186694

[32] de Mello MS, Oliveira AC. Overview of the actions to combat bacterial resistance in large hospitals. Rev Lat Am Enfermagem. 2021. 10.1590/1518-8345.3952.3407. 33852679 10.1590/1518-8345.3952.3407 PMC8040782

[33] McCallum H, Barlow N, Hone J. How should pathogen transmission be modelled? Trends Ecol Evol. 2001;16(6):295–300. 11369107 10.1016/s0169-5347(01)02144-9

[34] Doan TN, Kong DCM, Marshall C, Kirkpatrick CMJ, McBryde ES. Characterising the transmission dynamics of *Acinetobacter**baumannii* in intensive care units using hidden markov models. PLoS ONE. 2015;10(7):e0132037. 26131722 10.1371/journal.pone.0132037 PMC4489495

[35] Kirkeby C, Halasa T, Gussmann M, Toft N, Græsbøll K. Methods for estimating disease transmission rates: evaluating the precision of Poisson regression and two novel methods. Sci Rep. 2017;7(1):9496. 28842576 10.1038/s41598-017-09209-x PMC5572698

[36] Kojic EM, Darouiche RO. Candida infections of medical devices. Clin Microbiol Rev. 2004;17(2):255–67. 15084500 10.1128/CMR.17.2.255-267.2004 PMC387407

[37] Stewart PS, Costerton JW. Antibiotic resistance of bacteria in biofilms. Lancet. 2001;358(9276):135–8. 11463434 10.1016/s0140-6736(01)05321-1

[38] Cochran WL, McFeters GA, Stewart PS. Reduced susceptibility of thin *Pseudomonas**aeruginosa* biofilms to hydrogen peroxide and monochloramine. J Appl Microbiol. 2000;88(1):22–30. 10735239 10.1046/j.1365-2672.2000.00825.x

[39] Yu F, Cheng F, Yang Q, Wen Q, Li L. Monitoring the effect of microbial culture on cleaning and sanitizing of the external ventilator circuit. Cell Mol Biol. 2022;68(2):42–7. 35869724 10.14715/cmb/2022.68.2.6

[40] Francolini I, Donelli G. Prevention and control of biofilm-based medical-device-related infections. FEMS Immunol Med Microbiol. 2010;59(3):227–38. 20412300 10.1111/j.1574-695X.2010.00665.x

